# Bayesian hierarchical lasso Cox model: A 9-gene prognostic signature for overall survival in gastric cancer in an Asian population

**DOI:** 10.1371/journal.pone.0266805

**Published:** 2022-04-14

**Authors:** Jiadong Chu, Na Sun, Wei Hu, Xuanli Chen, Nengjun Yi, Yueping Shen

**Affiliations:** 1 Department of Biostatistics, School of Public Health, Medical College of Soochow University, Suzhou, P.R. China; 2 Department of Biostatistics, School of Public Health, University of Alabama at Birmingham, Birmingham, Alabama, United States of America; Shantou University Medical College, CHINA

## Abstract

**Objective:**

Gastric cancer (GC) is one of the most common tumour diseases worldwide and has poor survival, especially in the Asian population. Exploration based on biomarkers would be efficient for better diagnosis, prediction, and targeted therapy.

**Methods:**

Expression profiles were downloaded from the Gene Expression Omnibus (GEO) database. Survival-related genes were identified by gene set enrichment analysis (GSEA) and univariate Cox. Then, we applied a Bayesian hierarchical lasso Cox model for prognostic signature screening. Protein-protein interaction and Spearman analysis were performed. Kaplan–Meier and receiver operating characteristic (ROC) curve analysis were applied to evaluate the prediction performance. Multivariate Cox regression was used to identify prognostic factors, and a prognostic nomogram was constructed for clinical application.

**Results:**

With the Bayesian lasso Cox model, a 9-gene signature included *TNFRSF11A*, *NMNAT1*, *EIF5A*, *NOTCH3*, *TOR2A*, *E2F8*, *PSMA5*, *TPMT*, and *KIF11* was established to predict overall survival in GC. Protein-protein interaction analysis indicated that *E2F8* was likely related to *KIF11*. Kaplan-Meier analysis showed a significant difference between the high-risk and low-risk groups (P<0.001). Multivariate analysis demonstrated that the 9-gene signature was an independent predictor (HR = 2.609, 95% CI 2.017–3.370), and the C-index of the integrative model reached 0.75. Function enrichment analysis for different risk groups revealed the most significant enrichment pathway/term, including pyrimidine metabolism and respiratory electron transport chain.

**Conclusion:**

Our findings suggested that a novel prognostic model based on a 9-gene signature was developed to predict GC patients in high-risk and improve prediction performance. We hope our model could provide a reference for risk classification and clinical decision-making.

## Introduction

Gastric cancer (GC) has become a pervasive cancer worldwide and was responsible for over 1 million new cases and approximately 0.78 million deaths in 2018, making it the third leading cause of cancer death [1]. In addition, the incidence and diagnosis rates of GC in Asia were higher than those in other regions, especially among men [1]. Although constant improvements in therapy have been made, the survival rate of GC is still unsatisfactory, especially in the advanced stage [2]. With the development of high-throughput genome sequencing techniques, the exploration and application of various molecular biomarkers in GC would be efficient for better diagnosis, prediction, and targeted therapy [3]. Therefore, it is necessary and significant to establish a robust model based on genomic information for predicting prognosis. However, the occurrence and development of tumours are so complicated that considering a single gene to predict the survival of GC patients may not be accurate enough. Thus, it is feasible to construct a model by combining multiple genes with clinical characteristics to predict the survival prognosis of GC patients.

Further, identifying prognostic genes from high-dimensional data is critical, and several methods have been fully discussed [4]. Recently, Bayesian approaches have increased, thereby providing another option for variable selection and modelling based on high-dimensional survival data [5–11]. In previous research, Tang and Yi *et al*. proposed a novel Bayesian hierarchical Cox proportional hazards model (*i*.*e*., the spike-and-slab lasso Cox) for adapting high-dimensional molecular data [12]. Extensive simulation studies showed that the spike-and-slab lasso Cox outperformed other methods, such as lasso Cox. However, few studies apply the Bayesian model to specific tumours in practice.

This study aimed to apply the spike-and-slab lasso Cox for identifying potential prognostic genes using the GSE66229 dataset. We constructed a 9-gene signature and established an integrative prognostic model for predicting overall survival (OS) in GC patients. Model performance was assessed in an independent external validation set. Functional enrichment analysis for different risk groups was performed by GSEA. Then, an instructive nomogram was drawn for prediction in clinical application. This study not only enriches the practical application of Bayesian methods but also provides new ideas and references for clinical prognosis prediction modelling.

## Materials and methods

### Cancer database download and processing

The mRNA expression profile and clinical information were publicly downloaded from the Gene Expression Omnibus (GEO) (https://www.ncbi.nlm.nih.gov/geo/). The gene expression microarray dataset GSE66229 came from an Asian Cancer Research Group (ACRG) study that included 300 tumour tissue samples (mainly adenocarcinoma) and 100 normal tissue samples, and this was used as the training set in this study. Another Singapore cohort dataset, GSE15459, was an independent external validation set and included 182 tumour tissue samples. Both datasets were generated by the Affymetrix Human Genome U133 Plus 2.0 platform, and GSE66229 was normalized by robust multiarray average with the Affymetrix Power Tools package. GSE15459 was handled by Microarray Suite version 5.0 using Affymetrix default analysis settings and global scaling as a normalization method. Log2 transformation was utilized in this study.

### Gene Set Enrichment Analysis (GSEA)

In contrast to traditional analysis, GSEA was not limited to providing a clear threshold (e.g., log2FC) for differentially expressed genes but was focused on genes contributing to specific biological function gene sets [13]. It could avoid removing important genes with no statistically significant expression differences. In this study, samples from GSE66229 were divided into the tumour and non-tumour groups. Then, GSEA v4.1.0 was used to analyze gene data. Based on the Molecular Signatures Database, the gene sets of Kyoto Encyclopedia of Genes and Genomes (KEGG, c2.cp.kegg.v7.2.symbols.gmt) and Gene Ontology (GO, c5.go.v7.2.symbols.gmt) were set as reference datasets. Nom-P value<0.05 and false discovery rate (FDR) <0.25 were set as the cut-off values. In addition, function enrichment analysis based on the same settings was performed for different risk groups.

### Establishment of the prognostic signature

To screen prognostic genes, we split this process into two steps. First, univariate Cox regression was used to choose survival-related genes from the leading edge analysis in GSEA. Then, the spike-and-slab lasso Cox proposed by Tang and Yi *et al*. [12] was used to further identify prognostic genes. The spike-and-slab mixture double-exponential prior applied in this model was the key part and was expressed as follows:

$$\beta_{j}|\gamma_{j},s_{0},s_{1}∼(1-\gamma_{j})DE(\beta_{j}|0,s_{0})+\gamma_{j}DE(\beta_{j}|0,s_{1})$$ (1)

It had two positive value parameters, s_1_ and s_0_ (s_1_>s_0_>0), which need to be preset. s_0_ was chosen to be small and regarded as a “spike scale” for giving strong shrinkage on coefficient estimation, while s_1_ was set to be large so that it served as a “slab scale” for giving weak shrinkage on important variables. γ_j_ was the indicator variable that linked the scale parameters with the coefficients. The algorithm for fitting the spike-and-slab Cox model was called the expectation-maximization (EM) cyclic coordinate descent algorithm, which had a fast computing speed [12].

After filtering prognostic genes by the Bayesian hierarchical lasso Cox model, the risk score was calculated for each patient, and the formula was as follows:

$$Risk\;Score=\sum_{i=1}^{n}\left(\beta_{i}\times expression({gene}_{i})\right)$$ (2)

β_i_ represents the corresponding coefficient of a specific gene, and the expression () indicates the expression level of the corresponding gene. Next, samples were divided into high-risk or low-risk groups according to the median risk score. Kaplan-Meier and receiver operating characteristic (ROC) curve analysis were performed to assess the predictive effect. Also, we integrated the risk score and clinical factors into one model to build the final prognostic model and evaluated the performance by the C-index in the training set and validation set. Then, forest plots, nomogram plots, and calibration plots were used to demonstrate the main result fully.

### Correlation and validation analysis

To further explore possible associations, we firstly applied the Search Tool for the Retrieval of Interacting Genes (STRING, https://cn.string-db.org/) [14] to construct the Protein-Protein interaction (PPI) network based on the above genes. Then Spearman correlation analysis was performed to investigate the association at expression levels. On the other hand, the protein expression levels of the 9 genes were verified using the publicly Human Protein Altas (HPA) database (https://www.proteinatlas.org/) [15]. To better deal with the HPA data, we adopted the HPAanalyze package, a powerful tool for searching and analyzing the HPA database [16].

### External validation of prognostic model

We downloaded GSE15459 as an independent external validation set, which comprised the gene expression and clinical data of 182 samples. The risk scores of the GC patients were calculated and divided into high-risk and low-risk groups based on the median value. The robustness of this model was tested by Kaplan-Meier and ROC curve analysis.

### Implementation

Statistical analysis was performed based on R v4.0.2. bmlasso() was used for fitting the Bayesian hierarchical lasso Cox model, and cv.bh() was executed to select optimal s0 based on the predictive performance of the model. These functions are from the freely available R package BhGLM [17].

## Results

### Identification of survival-related genes

**Fig 1:**
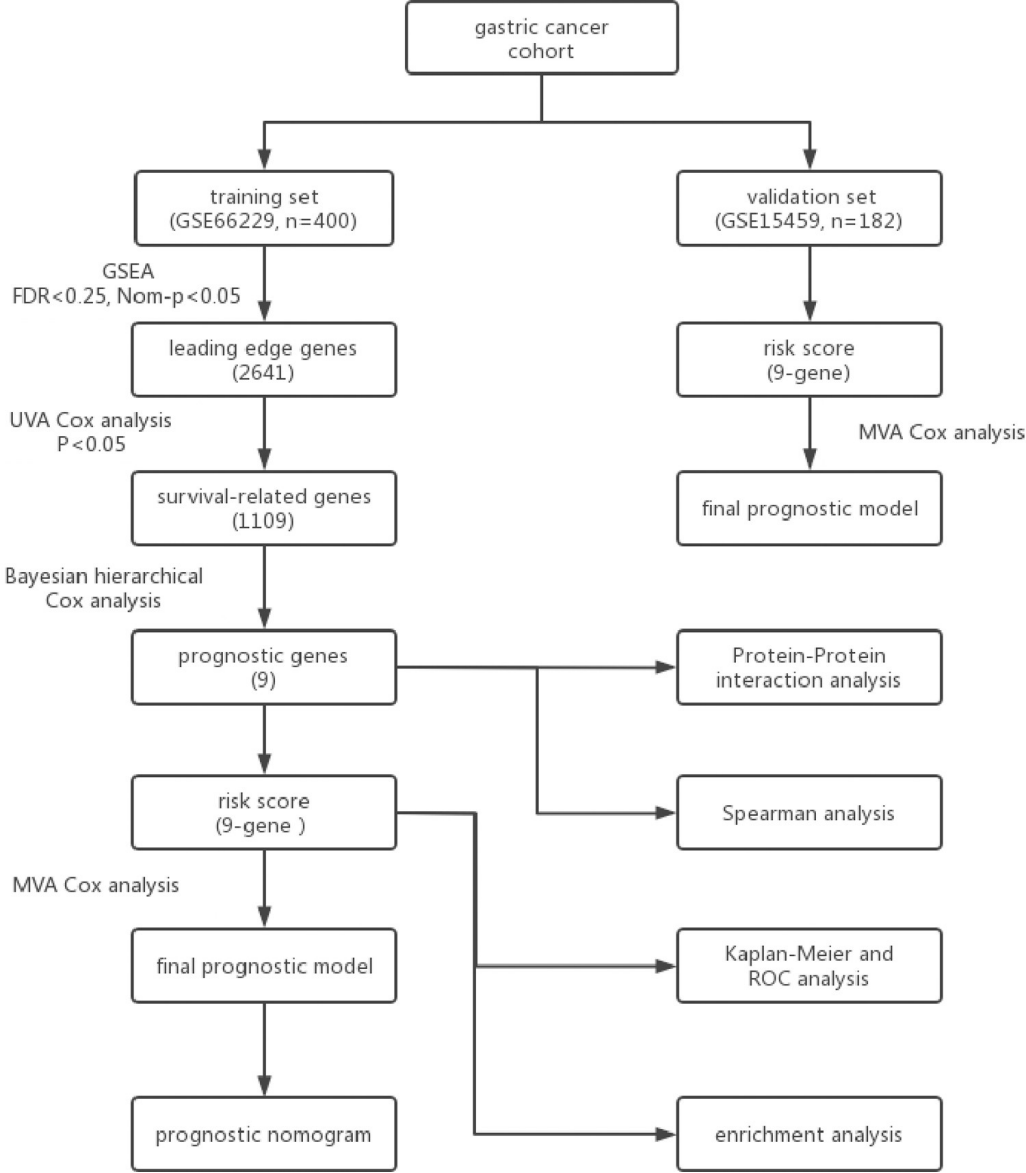
Schematic diagram of this study.

**Fig 2:**
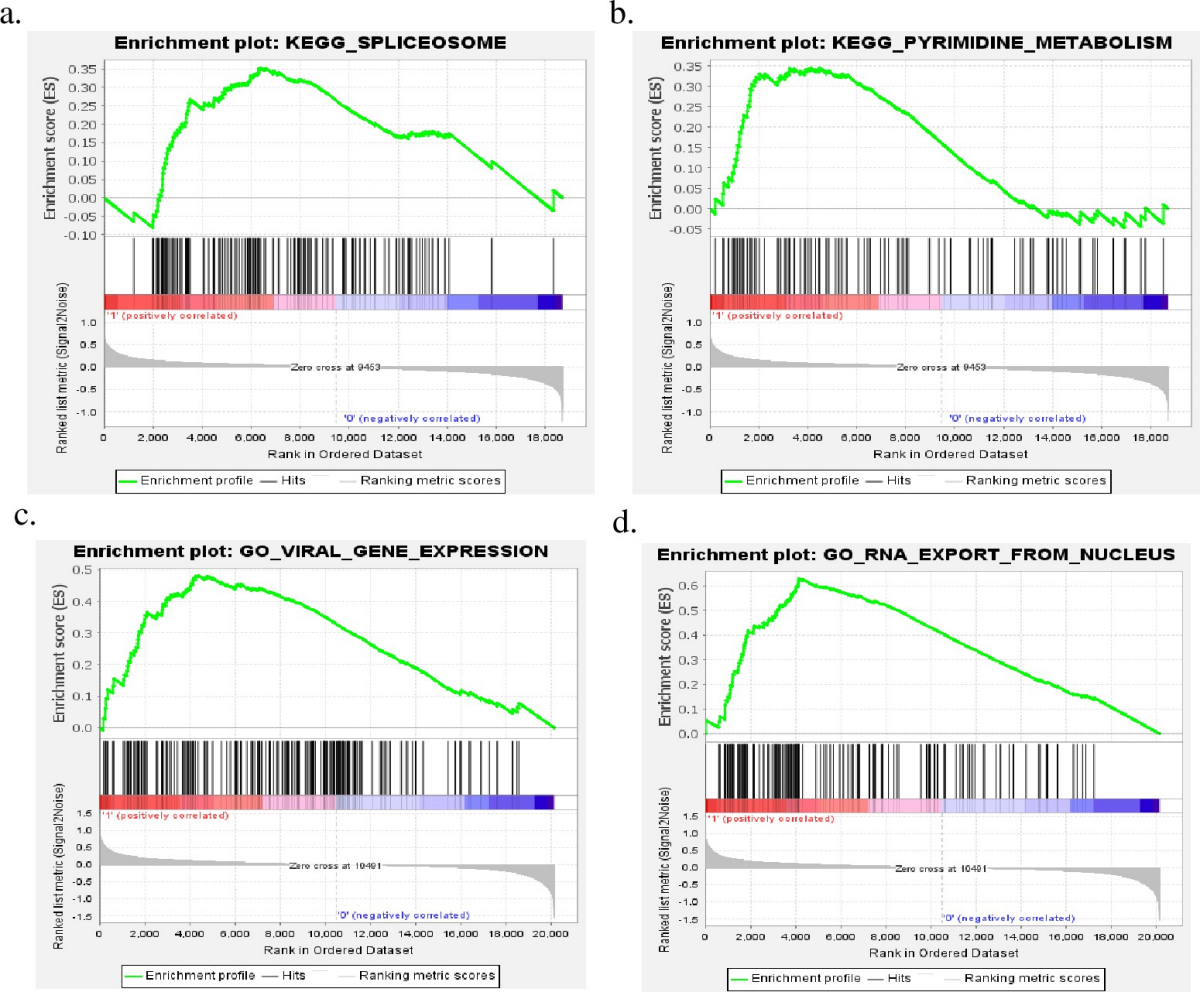
GSEA outcome of KEGG (a-b) and GO (c-d) with the largest absolute normalized enrichment size (NES).

To summarize this study more comprehensively, a schematic diagram was provided in Fig 1. GSE66229 had 400 samples (with a total of 20161 genes), including 300 cases and 100 normal samples. The GSEA results revealed that 331 genes involved in 15 pathways from KEGG and 2611 genes involved in 595 terms (biological process, molecular function, and cellular component) were filtered out with FDR<0.25 and Nom-P<0.05 (S1 Table). After removing duplicated genes, a total of 2641 genes were selected for the subsequent process. Among the above processes, pyrimidine metabolism (Nom-p = 0), spliceosome (Nom-p = 0.024), RNA export from the nucleus (Nom-p = 0), and viral gene expression (Nom-p = 0) from KEGG and GO based on the largest absolute normalized enrichment score (NES) were shown in Fig 2.

After excluding samples with missing values (i.e., survival time<1 month, survival outcome, pathological stage, age and sex), univariate Cox proportional hazards regression analysis was used to identify survival-related genes. Basic clinical characteristics were described completely in Table 1, and the results showed that 1109 genes were significantly correlated with OS at *P*<0.05.

**Table 1:** Clinical characteristics of patients with gastric cancer.

		Training set		Validation set	
		count	%	count	%
Survival status	Alive, 0	148	49.33	87	47.80
	Dead,1	152	50.67	95	52.20
Age	<65	161	53.67	75	41.21
	> = 65	139	46.33	107	58.79
Sex	female	101	33.67	66	36.26
	Male	199	66.33	116	63.74
Stage	I	30	10.00	31	17.03
	II	97	32.33	28	15.39
	III	96	32.00	66	36.26
	IV	77	25.67	57	31.32

### Bayesian hierarchical lasso Cox for screening final prognostic genes

The selection criterion of two parameters, s_1_ and s_0_, has been sufficiently discussed in a previous study [18]. The variety of the C-indexes of the survival model was sensitive to the change in s_0_ but less susceptible to s_1_. Therefore, we decided to fix s_1_ at 1 according to previous research. Regarding the value of s_0_, we first used the glmNet() function from BhGLM package to simulate 10-fold cross-validation repeated 10 times to obtain the stable penalty parameter λ (*i*.*e*., s_λ_) and then adjusted the value with a limited range from -0.04 to 0.06 with intervals of 0.01. Our goal was to find an optimal value that simultaneously made the C-index larger and deviance smaller. According to the results of 10-fold cross-validation with repeated 10 times, we ultimately decided to choose s_0_ = s_λ_-0.04 as an optimal value (Table 2). At the same time, the C-index and deviance of the constructed Bayesian hierarchical lasso Cox model were 0.684 (sd = 0.004) and 1582.454 (sd = 3.734), respectively. The C-index of traditional lasso Cox regression was 0.643 (sd = 0.011), which was lower than our Bayesian hierarchical lasso Cox model. Afterwards, we chose 9 prognostic genes whose coefficients were not zero, including tumour necrosis factor receptor superfamily member 11A (*TNFRSF11A*), nicotinamide nucleotide adenylyltransferase 1 (*NMNAT1*), eukaryotic translation initiation factor 5A (*EIF5A*), notch receptor 3 (*NOTCH3*), torsin family 2 member A (*TOR2A*), E2F transcription factor 8 (*E2F8*), proteasome 20S subunit alpha 5 (*PSMA5*), thiopurine S-methyltransferase (*TPMT*), and kinesin family member 11 (*KIF11*) (Table 3).

**Table 2:** Outcome of 10 times 10-fold cross-validation.

Method	C-index		Deviance	
	mean	sd	mean	sd
lasso	0.643	0.011	1602.125	5.641
s_λ_-0.04,1	0.684	0.004	1582.454	3.734
s_λ_-0.03,1	0.643	0.001	1601.508	1.258
s_λ_-0.02,1	0.642	0.001	1602.134	1.720
s_λ_-0.01,1	0.642	0.004	1603.394	5.236
s_λ_,1	0.636	0.009	1608.835	8.323
s_λ_+0.01,1	0.648	0.012	1600.793	6.695
s_λ_+0.02,1	0.653	0.012	1600.796	8.380
s_λ_+0.03,1	0.654	0.011	1605.169	11.075
s_λ_+0.04,1	0.657	0.011	1611.989	14.075
s_λ_+0.05,1	0.657	0.011	1623.413	16.959
s_λ_+0.06,1	0.655	0.012	1642.332	20.961

s_λ_ = 0.0400874379965632

**Table 3:** Bayesian hierarchical lasso Cox model of 9 genes associated with OS in GC patients.

Gene	Coefficient	HR
*TNFRSF11A*	-0.2277	0.7964
*NMNAT1*	-0.1997	0.8190
*EIF5A*	-0.1693	0.8443
*NOTCH3*	0.1525	1.1648
*TOR2A*	-0.1423	0.8673
*E2F8*	-0.0393	0.9615
*PSMA5*	-0.0363	0.9644
*TPMT*	-0.0214	0.9788
*KIF11*	-0.0060	0.9941

### PPI, Spearman correlation and validation analysis

**Fig 3:**
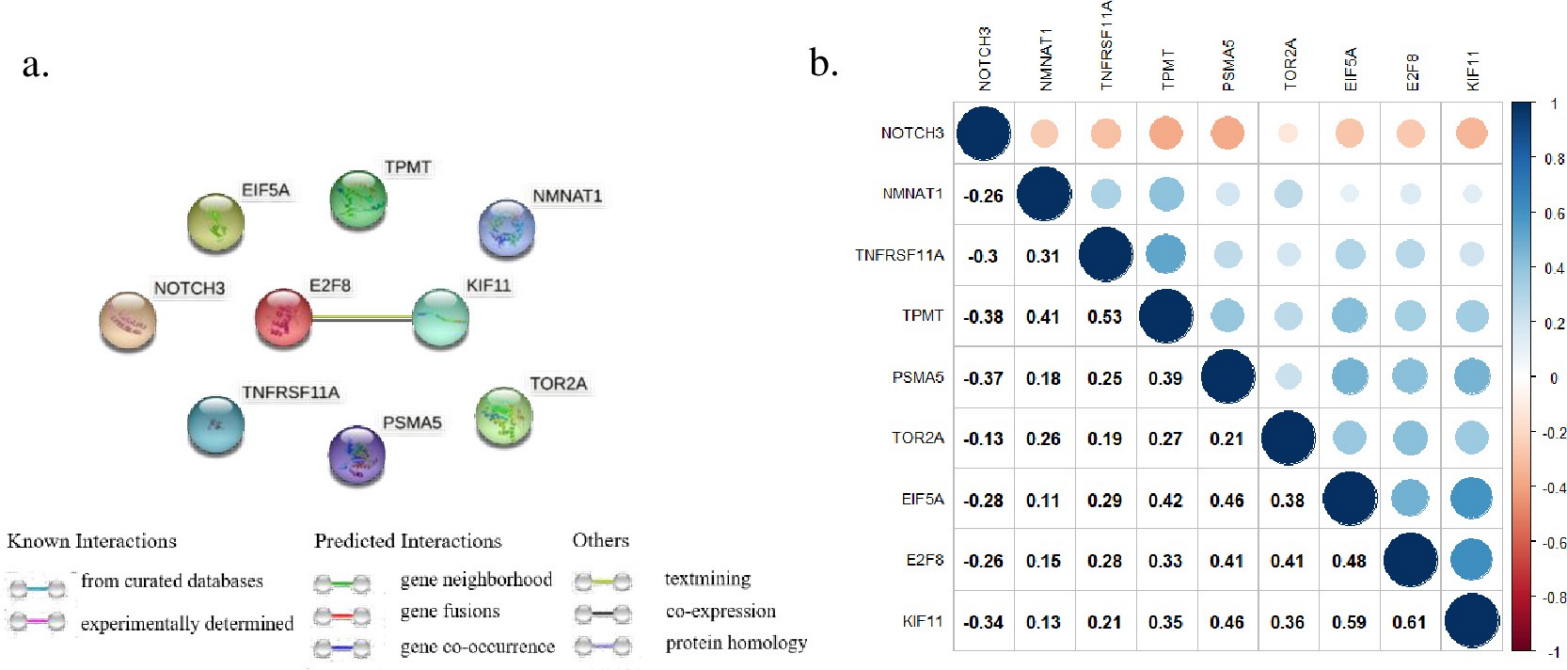
Interaction and correlation analysis. a) possible interactions in 9 genes based on STRING analysis. b) Spearman correlation plot of 9 genes based on expression profile.

PPI analysis was carried out to investigate possible inter-relationships of 9 genes (Fig 3A). According to the STRING database, there existed an interaction between *E2F8* and *KIF11*. Except for the association between *NMNAT1* and *EIF5A* (*P*>0.05), other pairs were statistically significant (*P*<0.05) (S2 Table). Notably, the expression of *NOTCH3* was negatively associated with other genes, and the strongest association came from the expression of *E2F8* and *KIF11* (r = 0.61) (Fig 3B). Further, the protein levels of these genes were also explored through the HPA database (S2 Fig). The result showed that three genes (*E2F8*, *KIF11*, *TPMT*) had been found to be highly expressed in GC tissue.

### Prognostic analysis of the 9-gene signature

We developed a prognostic signature based on the risk score constructed by the above 9 genes, and the formula of the risk score was given as follows: Riskscore = (-0.2277)*TNFRSF11A+(-0.1997)*NMNAT1+(-0.1693)*EIF5A+0.1525*NOTCH3+(-0.1423)*TOR2A+(-0.0393)*E2F8+(-0.0363)*PSMA5+(-0.0214)*TPMT+(-0.0060)*KIF11.

**Fig 4:**
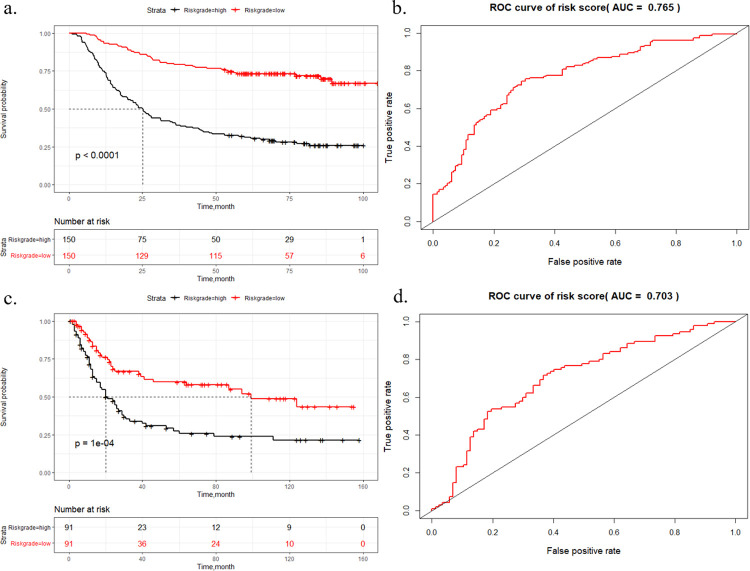
a) Kaplan-Meier curve of GSE66229 survival data for high-risk and low-risk groups with p<0.0001. b) The ROC curve of the risk score for predicting survival in the GSE66229 dataset. c) Kaplan-Meier curve of GSE15459 survival data for high-risk and low-risk groups with p = 0.0001. d) The ROC curve of the risk score for predicting survival in the GSE15459 dataset.

**Fig 5:**
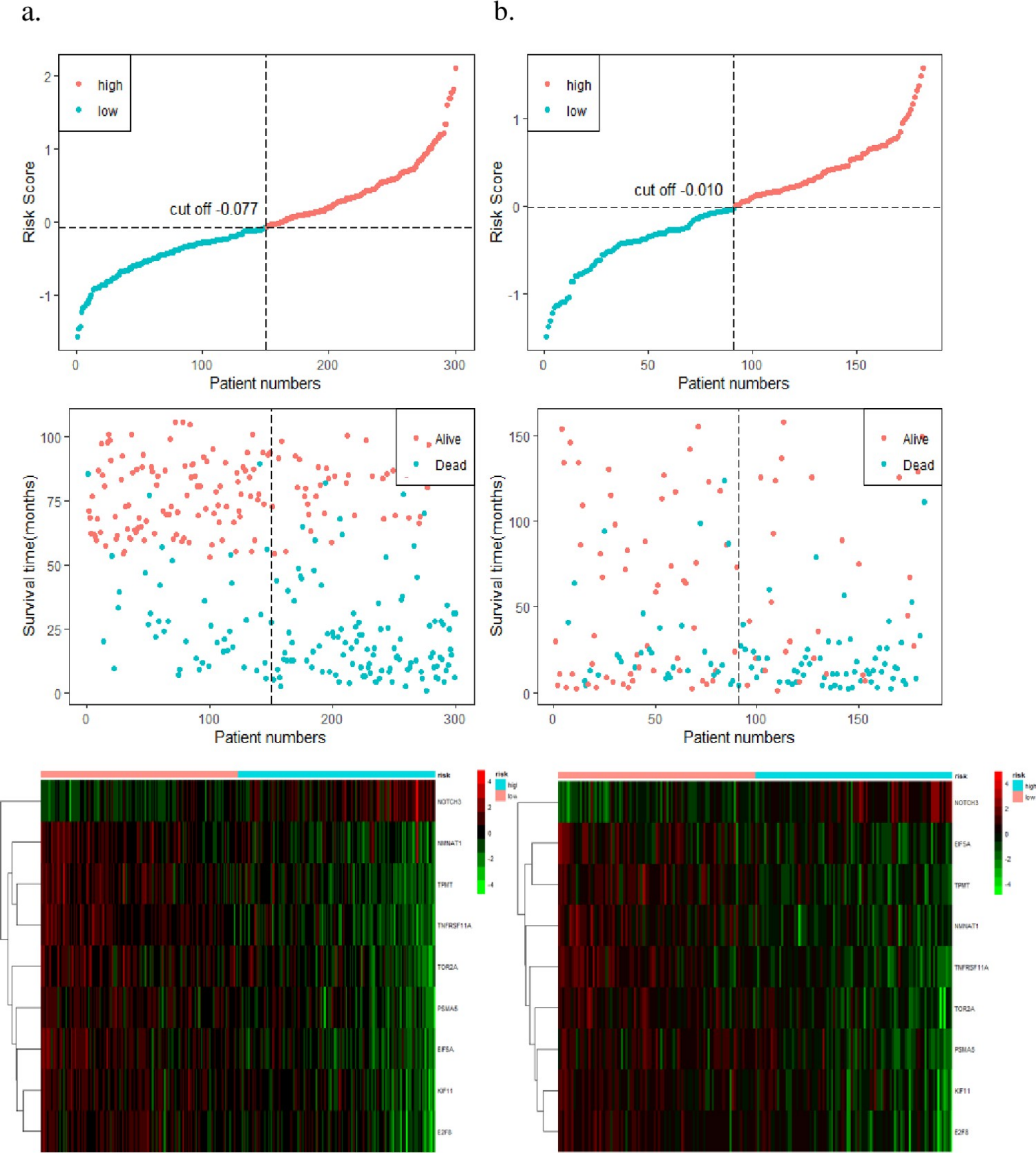
Risk score model based on 9 genes in the training and validation sets. a) The training set. b) The validation set. The top row shows the tendency of the risk score with the cut-off value in each dataset. The middle row shows the distribution of the survival status of patients in each dataset. The bottom row shows the mRNA expression of 9 genes based on the median risk score in each dataset.

After calculating the risk score for each patient, the median risk score (median = -0.077) was regarded as the cut-off value that stratified GC patients into low-risk and high-risk groups. Among them, the range of risk scores was [-1.569, 2.104]. The low-risk group was defined as [-1.569, -0.077), and the high-risk group was defined as [-0.077, 2.104]. The Kaplan-Meier survival analysis demonstrated a statistically significant difference between the low-risk and high-risk groups (*P*<0.0001, Fig 4A), and the AUC of the risk score was 0.765 (Fig 4B). The distribution of survival status is also shown in Fig 5A. As the risk score of the GC patients increased, the expression of the mRNAs in the high-risk group (*TNFRSF11A*, *NMNAT1*, *EIF5A*, *TOR2A*, *E2F8*, *PSMA5*, *TPMT*, *KIF11*) showed obvious downregulation, whereas the expression of mRNAs in the low-risk group (*NOTCH3*) was upregulated. Additionally, the Kaplan-Meier analysis of the expression of each gene is shown in S1 Fig.

### Function enrichment analysis

**Fig 6:**
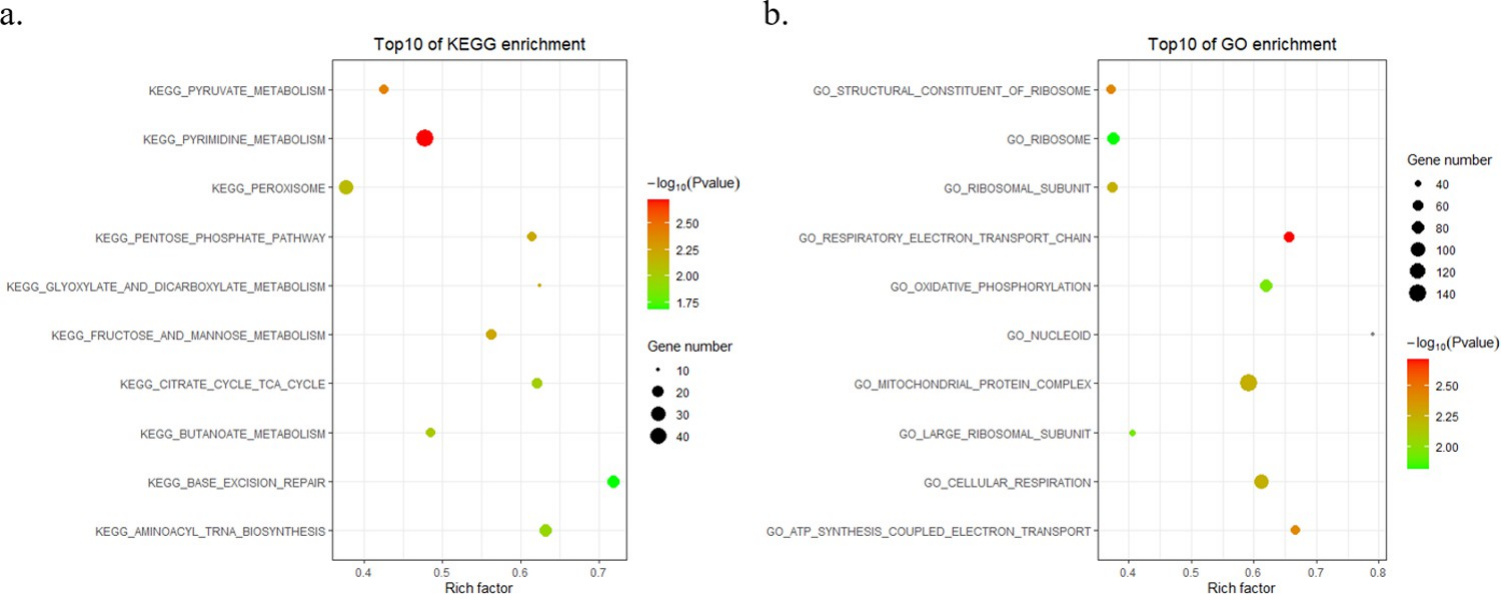
Functional enrichment analysis based on low-risk and high-risk groups. a) Top 10 enrichment pathways in KEGG based on the largest absolute NES. b) Top 10 enriched GO pathways based on the largest absolute NES.

To elucidate the possible biological terms or pathways associated with the risk score, we also utilized GSEA to perform GO and KEGG pathway analyses based on differentially expressed risk genes (DRGs) between the low-risk and high-risk groups. As shown in the chart, we intuitively discovered that DRGs were enriched mainly in particular molecular terms/pathways, such as pyrimidine metabolism and respiratory electron transport chain (Fig 6A and 6B).

### Combining 9-gene signature with clinical characteristics

**Fig 7:**
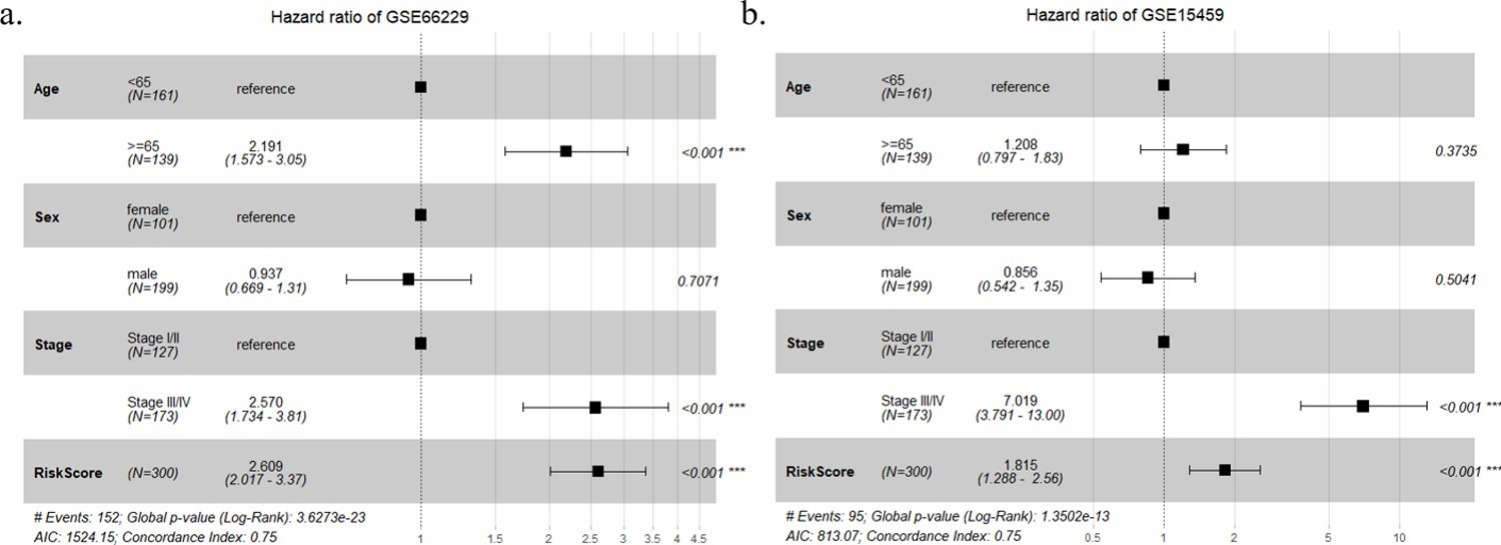
Integrative prognostic models combining the risk score and clinical factors in GC patients. a) Forest plot combining the risk score with clinical factors (age, sex, stage) in the training set. b) Forest plot combining the risk score with clinical factors (age, sex, stage) in the validation set.

**Fig 8:**
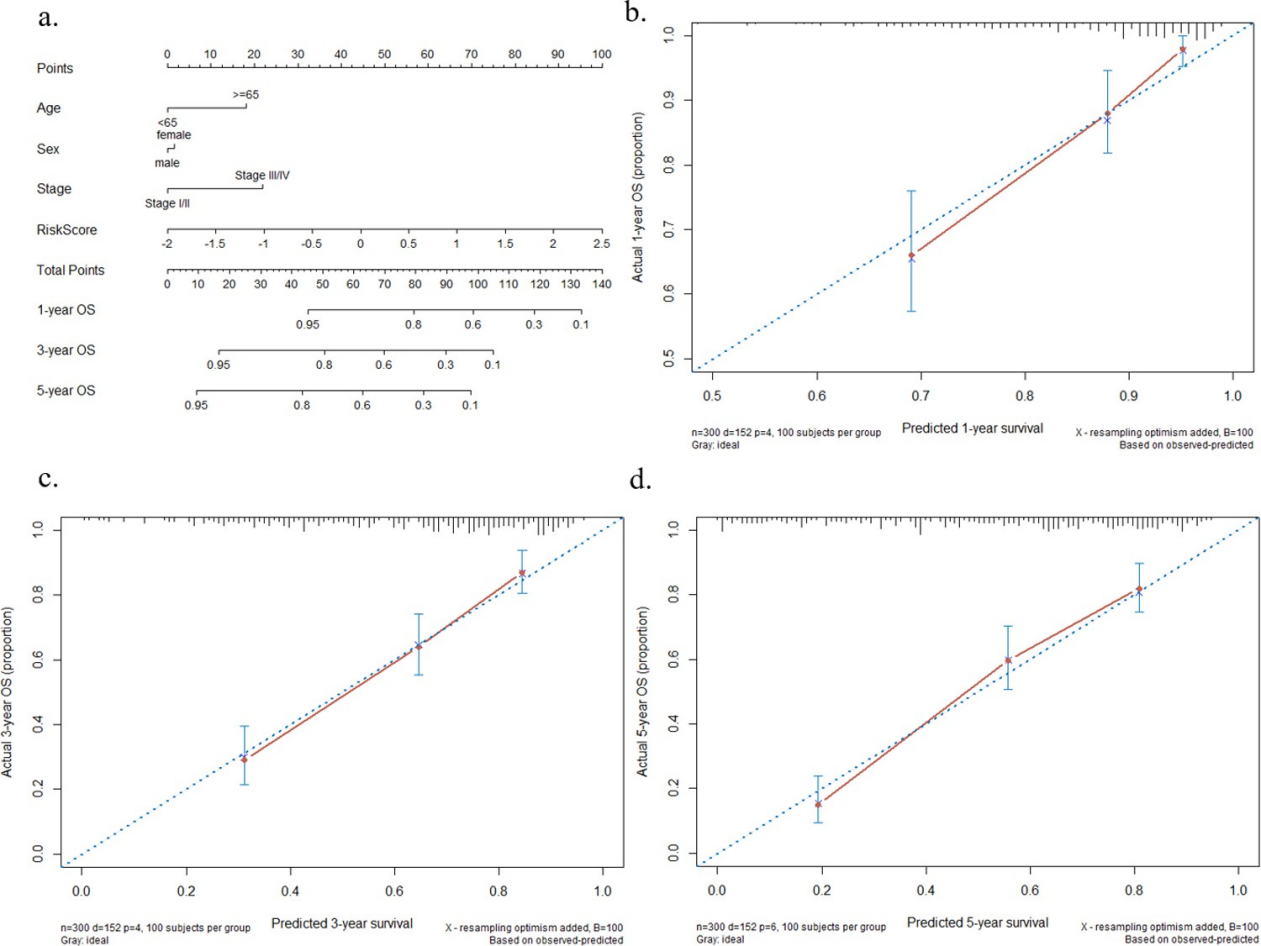
Nomogram and calibration plots for the prognostic model. a) Nomogram plotted by gene signature and clinical factors. b-d) Calibration plots demonstrating the consistency between predicted and observed 1-year, 3-year, and 5-year survival outcomes.

Next, we integrated the gene signature plus several clinical factors, including age, sex and stage, into a super prognostic model to completely predict the OS of GC patients. After integration, the C-index of the final prognostic model reached 0.75, in contrast to the model that considered only clinical factors (C-index = 0.686) (Fig 7A). The HR of the risk score was 2.609, 95% CI: 2.017–3.370. Finally, considering the application of clinical practice, we utilized a nomogram to predict the survival probability of GC patients (Fig 8A). Moreover, calibration plots were used to illustrate the stability of the nomogram in predicting 1-year, 3-year, or 5-year OS (Fig 8B–8D).

### Independent external validation in GSE15459

To assess the robustness of the 9-gene prognostic signature, we selected GSE15459 as an independent external validation set. Similar to the training set, Kaplan-Meier analysis indicated that low-risk patients had longer survival times than high-risk patients (*P* = 0.0001, Fig 4C), and the overall AUC of the risk score was 0.703 (Fig 4D). The distribution between the risk score and survival status is displayed in Fig 5B. We observed that the expression levels between different risk groups were similar to those in the training set, which further verified the accuracy of our results. After integrating clinical characteristics, the C-index of the final prognostic model also increased to 0.75, in contrast to the model with only clinical characteristics (C-index = 0.696). Multivariate analysis demonstrated that the risk score could be a stable prognostic factor for the prediction of OS (HR = 1.815, 95% CI: 1.288–2.560, *P*<0.001) (Fig 7B).

## Discussion

As one of the most common tumour diseases, GC has a relatively high incidence and mortality rate, especially in Asia [1]. Reliable markers could be applied as the vital indicator for GC risk stratification and following treatment. Compared to the traditional model with clinical characteristics, the prognostic model combining biomarkers would be more powerful for prediction performance, especially in the current era of precision medicine. However, the high-dimensional feature of genetic data makes it difficult to model directly, and some effective statistical methods are needed, such as PCA, ridge, and lasso [4]. In this study, we used a Bayesian approach (also known as spike-and-slab lasso Cox) to screen target genes [12]. This method integrated mainly the penalized lasso and Bayesian variable selection, which was superior to lasso. First, this Bayesian model could achieve the same function of variable selection as lasso Cox. Second, compared with lasso Cox giving the same penalty parameter to all coefficients, spike-and-slab lasso Cox could achieve more flexibility. In other words, it could selectively shrink different predictors based on different scales from the data (*i*.*e*., giving relatively small shrinkage to those predictors with large effect and giving strong shrinkage to irrelevant or weak predictors at the same time). Therefore, to some extent, this Bayesian model could reduce the estimation bias of lasso Cox. Our study also demonstrated that the cross-validated C-index of the Bayesian model was better than the traditional lasso (0.684 vs 0.643).

After choosing the optimal model, 9 prognostic genes with non-zero coefficients were selected. Next, we established the risk score index based on these genes, and Kaplan-Meier analysis demonstrated that GC patients in the high-risk group (> = -0.077) had a shorter survival time than those in the low-risk group (<-0.077). Additionally, we tested the predictive power of the 9-gene signature alone, and the results showed that it was a great predictor for OS (AUC = 0.765 in the training set; AUC = 0.703 in the validation set). Compared to a 4-gene signature reported by a previous study, our signature acquired a higher level (AUC for OS, 0.765 vs 0.684) [19]. Furthermore, we integrated the risk score with clinical factors (age, sex, pathological stage) to construct a multivariate Cox regression model. The results proved that this risk signature could be an independent prediction marker for GC. The C-index of the integrative model reached 0.75 in both the training set and validation set, which showed the reliable performance of our model. Finally, considering the possible clinical application, a nomogram was provided to visually predict the GC patients’ survival. By evaluating 1-year, 3-year, and 5-year OS calibration curves, the drawn nomogram had a relatively high accuracy of prediction.

Through the Bayesian hierarchical lasso Cox model, the 9 genes we found were also the focus of other basic researches or population studies. *NOTCH3*, a member of the NOTCH family, is involved in the NOTCH signalling pathway, which is regarded as one of the key pathways constituting the stem cell signalling network [20]. Our results demonstrated that the increased expression of *NOTCH3* was associated with poor OS among GC patients, which was consistent with previous studies [21, 22]. *TNFRSF11A*, also regarded as the receptor activator of NF-κB (RANK), can activate several pathways, such as NF-κB, JNK, ERK, p38, and Akt/PKB, and was reported to be a novel and frequent target for de novo methylation in gliomas [23]. Our study showed that the expression of *TNFRSF11A* was positively associated with survival (HR = 0.7964<1), which was consistent with another study [24]. In population-based studies, *TPMT*, *TOR2A*, *KIF11*, and *EIF5A* were reported to be associated with prognosis in other tumours, such as childhood acute lymphoblastic leukaemia [25, 26], ovarian cancer [27, 28], breast cancer [29], and colorectal cancer [30].

*NMNAT1* is involved in the NAD+ salvage/recycling pathway, which is crucial for maintaining the functions of a wide variety of NAD+-dependent enzymes in the cytoplasm and nucleus [31]. Knockdown of *NMNAT1* enhanced rRNA transcription, which might facilitate increased ribosome biogenesis and tumour development [32]. However, to our knowledge, for the *NMNAT1* signature, there have been no relevant studies based on populations. Our study revealed that high expression of *NMNAT1* was associated with lower mortality risk and higher OS than low expression. *E2F8* is a member of the E2F family of transcription factors that regulates various cellular functions related to the cell cycle and apoptosis [33]. *E2F8* is considered to be a kind of transcriptional repressor that is similar to *E2F7* in that it can inhibit E2F-driven promoters [34]. A previous study found that the increased expression of E2F family members (*E2F2*, *E2F5*, *E2F6*, and *E2F7*) was significantly associated with favourable OS in GC [35]. Our research further revealed that increased expression of *E2F8* was also associated with favourable prognosis.

Notably, some previously reported genes, such as *PSMA5* and *KIF11*, can be treated as potential therapeutic targets for tumours [36, 37]. Previous studies demonstrated that the upregulation of *PSMA5*, by activating the key Nrf2/ARE signalling pathway, played a critical role in the mechanism of inducing tumour cell apoptosis caused by combined chemotherapy regimens [38, 39]. *KIF11* silencing induced chromosome instability (CIN), which might contribute to cancer development and progression [40]. However, other studies showed that the suppression of *PSMA5* could strengthen the sensitivity of myeloma to bortezomib [41]. Apigenin induced apoptosis in prostate cancer cells, which was accompanied by the downregulated expression of *PSMA5* [42]. These studies suggested that the same gene might involve different mechanisms in different tumour types.

In general, our study used a Bayesian hierarchical lasso Cox model to screen a prognosis-related gene signature. It was the first to apply this Bayesian approach to construct prognosis-related models in GC. Notably, there might be some restrictions regarding generalizability to populations in other regions because this model we built was based on the Asian population. Moreover, our analysis focused mainly on mRNA expression data, but other molecular types, such as microRNA, CNV, or methylation, might contain important prognostic information in GC. Therefore, in further research, we would consider establishing a more generalized model that combines different molecular data for better prediction based on our Bayesian approach. Finally, although the 9-gene signature was explored by statistical analysis or database validation, we expect to further verify these genes by in vitro/vivo experiments in the future study.

## Conclusion

Our research confirmed that the Bayesian hierarchical lasso Cox model had great prediction power than the traditional Cox model. Based on this Bayesian approach, we proposed a 9-gene prognostic signature as an independent predictor for the overall survival of GC patients. Finally, combined with clinical characteristics, a comprehensive nomogram was provided for clinical application. Overall, our study offers certain reference significance for clinical prognosis prediction in GC.

## Supporting information

S1 Table Leading edge subset outcome of GSEA. (XLSX)

S2 Table The Spearman correlation test among 9 genes from GSE66229. (DOCX)

S1 Fig Kaplan-Meier analysis of each gene based on median expression level for overall survival. (DOCX)

S2 Fig The immunohistochemistry images of nine genes in normal and tumour tissues derived from the Human Protein Altas (HPA) database. (DOCX)
