# Supplementary material for: Bayesian hierarchical lasso Cox model: A 9-gene prognostic signature for overall survival in gastric cancer in an Asian population
Source: PLoS One. 2022 Apr 14;17(4):e0266805. doi: 10.1371/journal.pone.0266805 (PMC9009599; doi:10.1371/journal.pone.0266805)
Supplement: S2 Table — (DOCX) [file pone.0266805.s002.docx]

S2 Table. The Spearman correlation test among 9 genes from GSE66229.

| **Gene1** | **Gene2** | **Estimate** | ***P* value** |
| --- | --- | --- | --- |
| *NMNAT1* | *EIF5A* | 0.11 | 6.14E-02 |
| *NMNAT1* | *PSMA5* | 0.18 | 1.53E-03 |
| *NMNAT1* | *NOTCH3* | -0.26 | 7.40E-06 |
| *NMNAT1* | *TPMT* | 0.41 | 2.72E-13 |
| *NMNAT1* | *KIF11* | 0.13 | 2.75E-02 |
| *NMNAT1* | *E2F8* | 0.15 | 1.16E-02 |
| *NMNAT1* | *TOR2A* | 0.26 | 6.40E-06 |
| *NMNAT1* | *TNFRSF11A* | 0.31 | 2.87E-08 |
| *EIF5A* | *PSMA5* | 0.46 | <2.2E-16 |
| *EIF5A* | *NOTCH3* | -0.28 | 8.82E-07 |
| *EIF5A* | *TPMT* | 0.42 | 1.58E-14 |
| *EIF5A* | *KIF11* | 0.59 | <2.2E-16 |
| *EIF5A* | *E2F8* | 0.48 | <2.2E-16 |
| *EIF5A* | *TOR2A* | 0.38 | 1.85E-11 |
| *EIF5A* | *TNFRSF11A* | 0.29 | 2.21E-07 |
| *PSMA5* | *NOTCH3* | -0.37 | 3.56E-11 |
| *PSMA5* | *TPMT* | 0.39 | 4.68E-12 |
| *PSMA5* | *KIF11* | 0.46 | <2.2E-16 |
| *PSMA5* | *E2F8* | 0.41 | 7.59E-14 |
| *PSMA5* | *TOR2A* | 0.21 | 2.06E-04 |
| *PSMA5* | *TNFRSF11A* | 0.25 | 9.78E-06 |
| *NOTCH3* | *TPMT* | -0.38 | 1.36E-11 |
| *NOTCH3* | *KIF11* | -0.34 | 2.02E-09 |
| *NOTCH3* | *E2F8* | -0.26 | 4.08E-06 |
| *NOTCH3* | *TOR2A* | -0.13 | 2.47E-02 |
| *NOTCH3* | *TNFRSF11A* | -0.30 | 1.37E-07 |
| *TPMT* | *KIF11* | 0.35 | 7.43E-10 |
| *TPMT* | *E2F8* | 0.33 | 2.68E-09 |
| *TPMT* | *TOR2A* | 0.27 | 2.63E-06 |
| *TPMT* | *TNFRSF11A* | 0.53 | <2.2E-16 |
| *KIF11* | *E2F8* | 0.61 | <2.2E-16 |
| *KIF11* | *TOR2A* | 0.36 | 9.29E-11 |
| *KIF11* | *TNFRSF11A* | 0.21 | 2.93E-04 |
| *E2F8* | *TOR2A* | 0.41 | 1.19E-13 |
| *E2F8* | *TNFRSF11A* | 0.28 | 1.13E-06 |
| *TOR2A* | *TNFRSF11A* | 0.19 | 1.17E-03 |
