# Supplementary figures and images for: Bayesian hierarchical lasso Cox model: A 9-gene prognostic signature for overall survival in gastric cancer in an Asian population
Source: PLoS One. 2022 Apr 14;17(4):e0266805. doi: 10.1371/journal.pone.0266805 (PMC9009599; doi:10.1371/journal.pone.0266805)

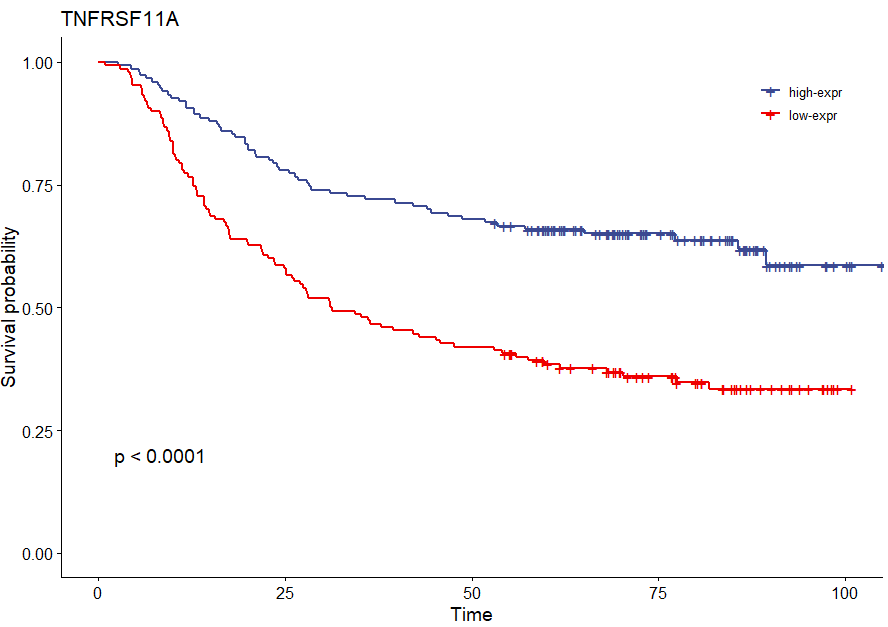

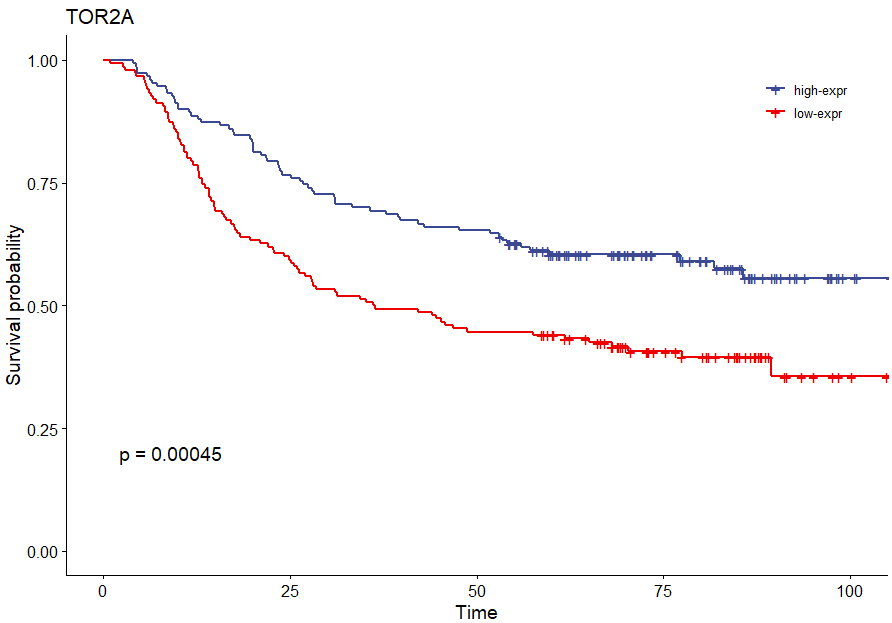

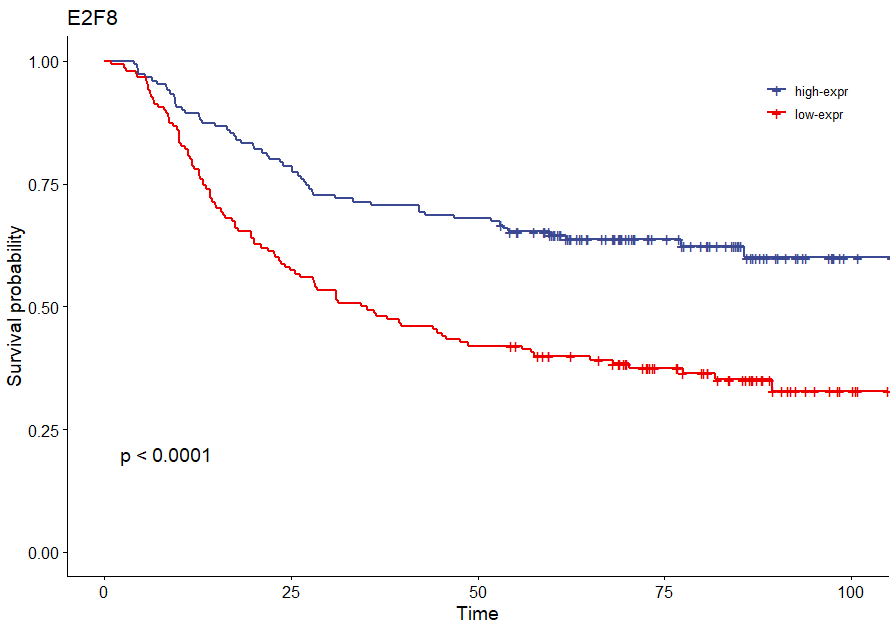

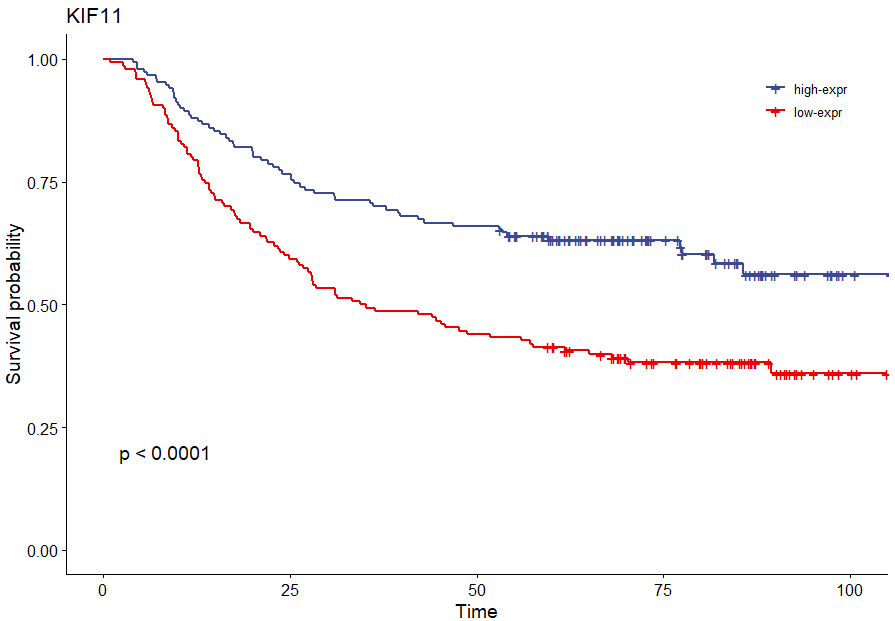

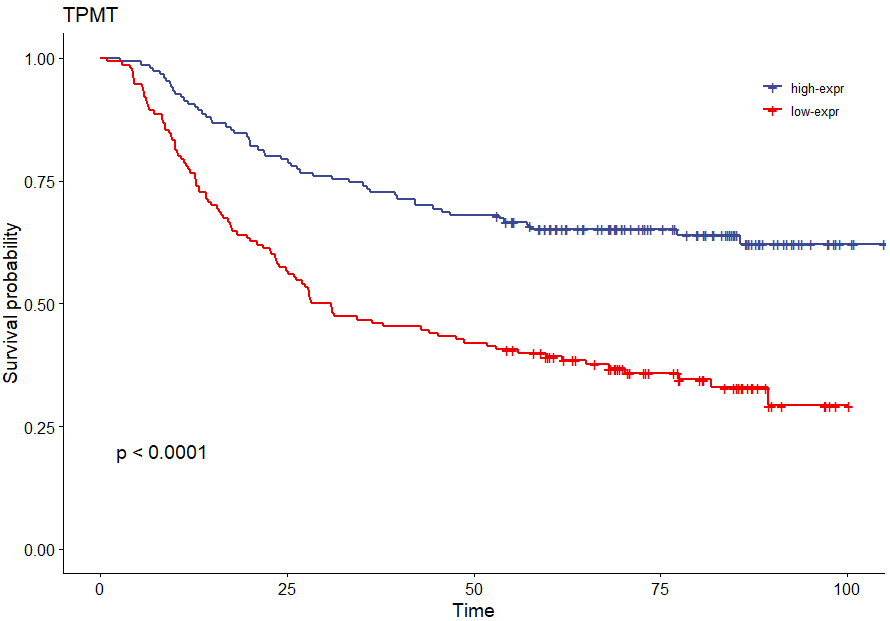

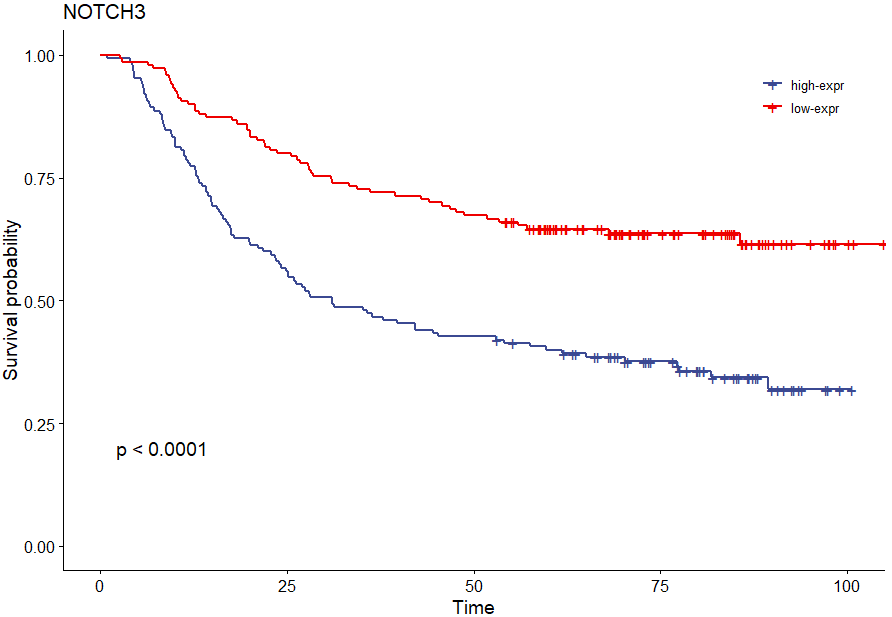

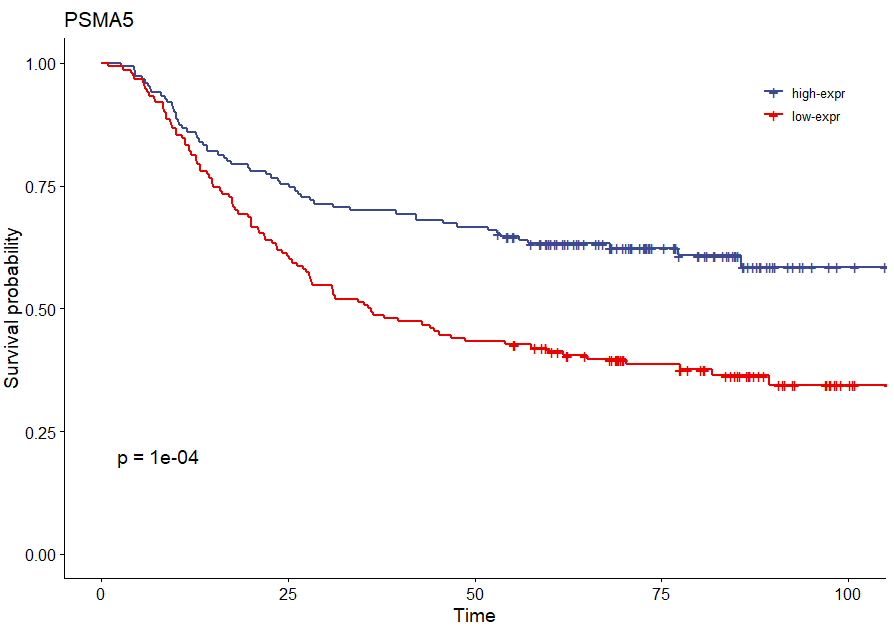

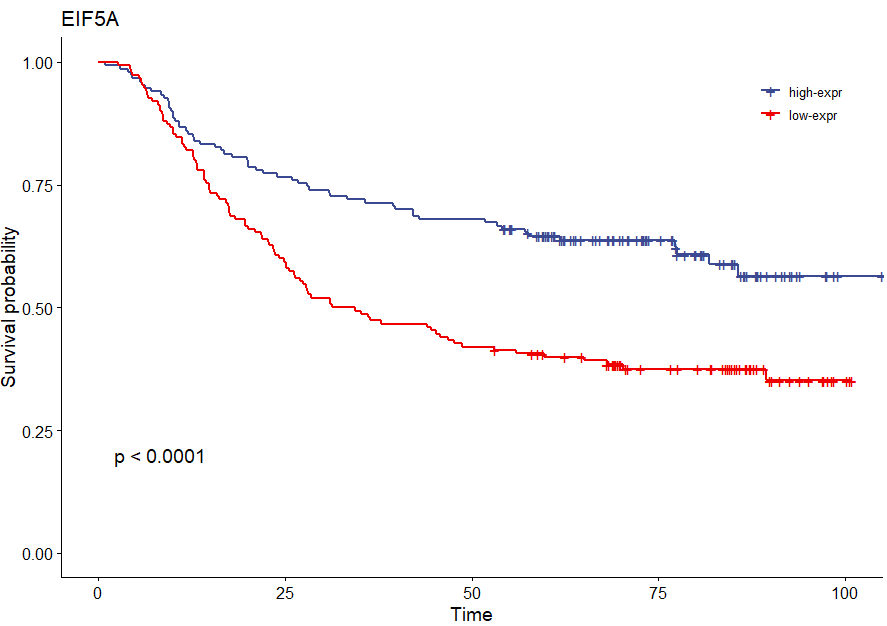

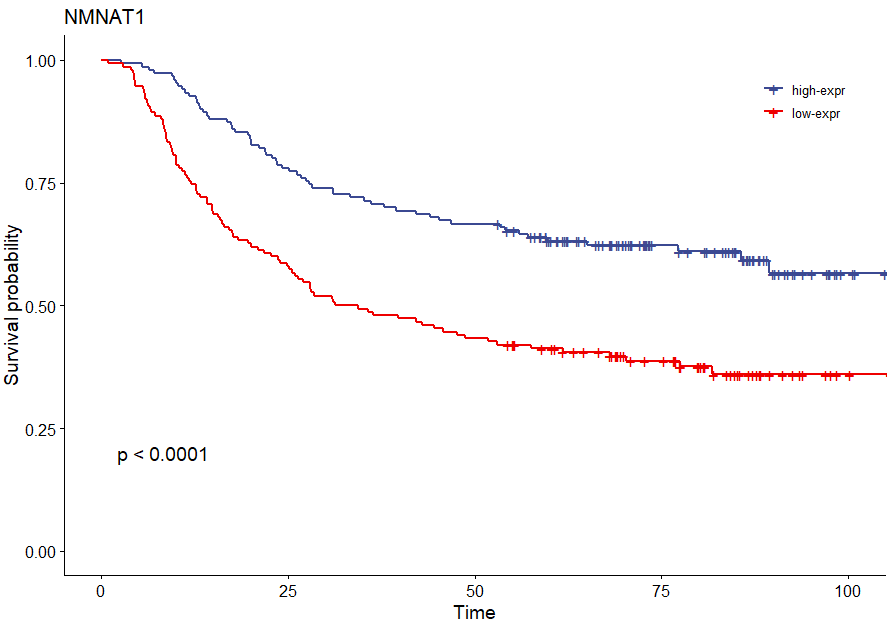


**S1 Fig**. Kaplan-Meier analysis of each gene based on median expression level for overall survival.

Supplement: S1 Fig — (DOCX) [file pone.0266805.s003.docx]
