# Supplementary material for: Bayesian hierarchical lasso Cox model: A 9-gene prognostic signature for overall survival in gastric cancer in an Asian population
Source: PLoS One. 2022 Apr 14;17(4):e0266805. doi: 10.1371/journal.pone.0266805 (PMC9009599; doi:10.1371/journal.pone.0266805)
Supplement: S2 Fig — (DOCX) [file pone.0266805.s004.docx]

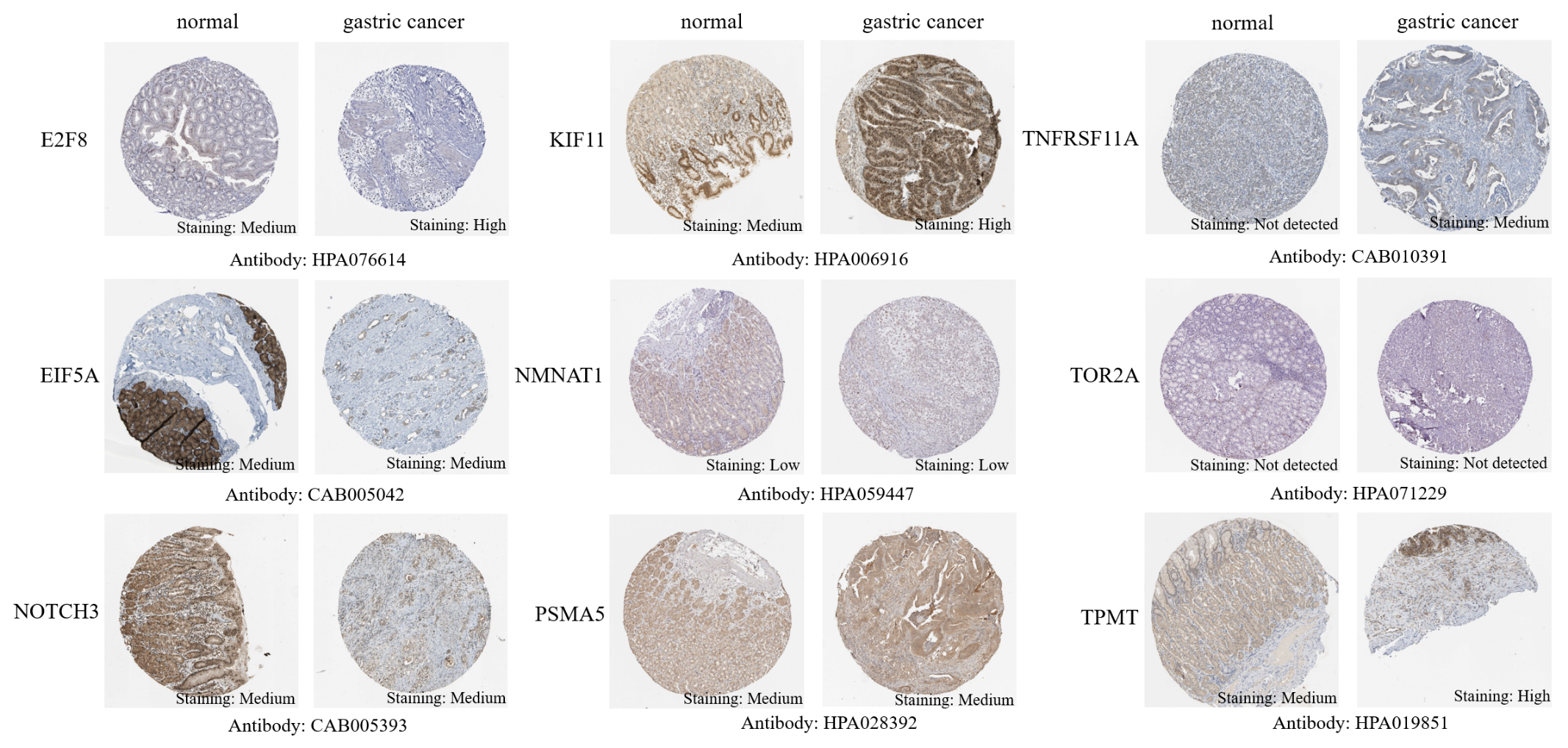


**S2 Fig**. The immunohistochemistry images of nine genes (*E2F8, EIF5A, NOTCH3, KIF11, NMNAT1, PSMA5, TNFRSF11A, TOR2A, TPMT*) in normal and tumour tissues derived from the Human Protein Altas (HPA) database.
